# Twenty years of Mediator complex structural studies

**DOI:** 10.1042/BST20180608

**Published:** 2019-02-07

**Authors:** Alexis Verger, Didier Monté, Vincent Villeret

**Affiliations:** UMR 8576, Unité de Glycobiologie Structurale et Fonctionnelle (UGSF), CNRS, Univ. Lille, F-59000 Lille, France

**Keywords:** Cryo-EM, Mediator complex, structural biology, transcription

## Abstract

Mediator is a large multiprotein complex conserved in all eukaryotes that plays an essential role in transcriptional regulation. Mediator comprises 25 subunits in yeast and 30 subunits in humans that form three main modules and a separable four-subunit kinase module. For nearly 20 years, because of its size and complexity, Mediator has posed a formidable challenge to structural biologists. The first two-dimensional electron microscopy (EM) projection map of Mediator leading to the canonical view of its division in three topological modules named Head, Middle and Tail, was published in 1999. Within the last few years, optimization of Mediator purification combined with technical and methodological advances in cryo-electron microscopy (cryo-EM) have revealed unprecedented details of Mediator subunit organization, interactions with RNA polymerase II and parts of its core structure at high resolution. To celebrate the twentieth anniversary of the first Mediator EM reconstruction, we look back on the structural studies of Mediator complex from a historical perspective and discuss them in the light of our current understanding of its role in transcriptional regulation.

## Introduction

The Mediator complex was initially identified in yeast in the early 1990s independently by the Kornberg and the Young laboratories. The Kornberg laboratory isolated Mediator biochemically from a distinct crude yeast fraction that stimulated activator-dependent transcription *in vitro* using partially purified proteins [1,2]. The Young laboratory used yeast genetic screens and identified the first Mediator genes as suppressors of truncations of the Carboxy-terminal domain (CTD) of RNA polymerase II (RNA Pol II). The product of four dominants suppressors termed Srb2, Srb4, Srb5 and Srb6 (Srb, suppressor of RNA polymerase B [3]) were shown to be part of a high molecular mass multisubunit complex that was tightly bound to the RNA Pol II [4]. An activity was soon after isolated that stimulated transcription *in vitro* in a form of a 20-subunit complex including Srb2, Srb4, Srb5 and Srb6 [5] (reviewed in [6]). Complexes with similar activities were subsequently purified in metazoans by many laboratories (reviewed in [7]). At first, it was unclear whether these complexes were all related to the yeast Mediator but comparative genomics [8,9] and multidimensional protein identification technology (MudPIT) [10] identified a set of consensus Mediator subunits conserved in all eukaryotes and a unified nomenclature was adopted in 2004 [11].

**Figure 1.**
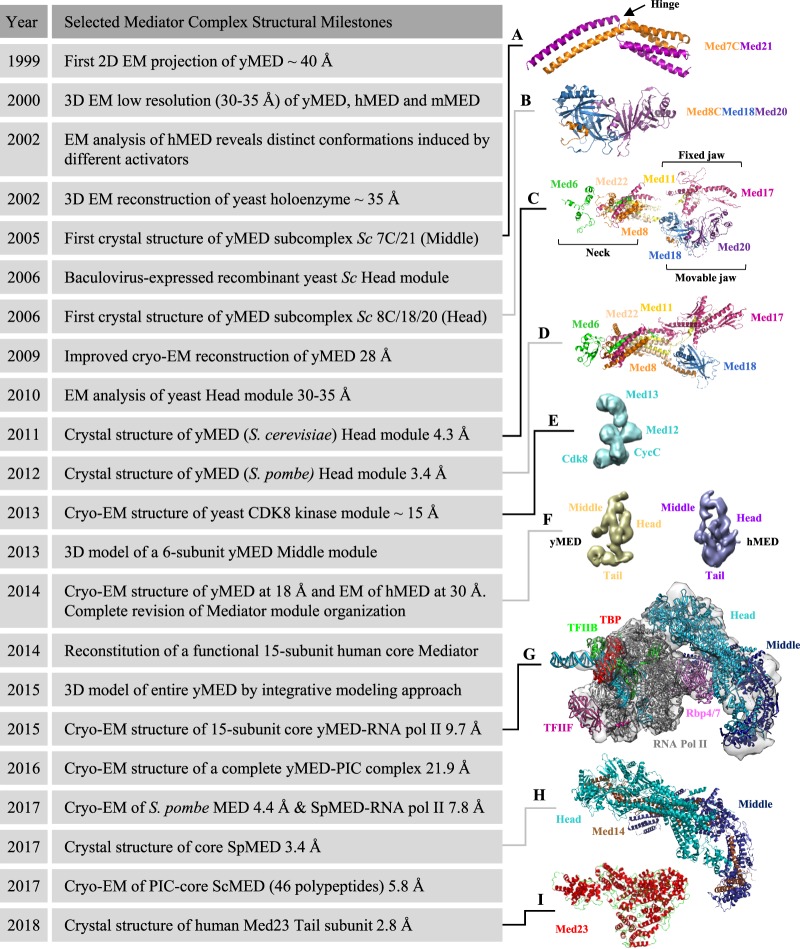
Timeline of selected milestones in Mediator complex structural studies. From the top down, the depicted structures are (**A**) *S. cerevisiae* Med7C/Med21 complex (protein data bank (PDB) 1YKH [42]). The conserved flexible hinge is indicated. (**B**) *S. cerevisiae* Med8C/18/20 submodule (PDB 2HZS [43]), (**C**) *S. cerevisiae* Head module (PDB 3RJ1 [53]). The three major domains described initially in 2011 (fixed jaw, movable jaw and neck) are indicated. (**D**) *S. pombe* Head module (PDB 4H63 [55]), (**E**) *S. cerevisiae* CDK8 kinase module (electron microscopy data bank (EMD)-5588 [99]), (**F**) *S. cerevisiae* (EMD-2634) and *Homo sapiens* (EMD-2635) [57] Mediator complex, (**G**) *S. cerevisiae* RNA Pol II–core Mediator transcription initiation complex (EMD-2786 [48]), (**H**) *S. pombe* core Mediator (PDB 5N9J [47]) and (**I**) *Homo sapiens* MED23 subunit (PDB 6H02 [72]). For details, refer to the text. For a complete collection of structural data on Mediator, see Supplementary Table S1 adapted from [28] with permission from Elsevier. Figures were prepared with PyMol [116] or UCSF Chimera [117]. y, yeast; h, human; m, murine; Sc, *Saccharomyces cerevisiae*, Sp, *Schizosaccharomyces pombe*.

Mediator acts as a physical and functional bridge between DNA-binding transcription factors and the transcription machinery. It regulates gene expression at multiple stages of transcription, from promoting assembly of the preinitiation complex (PIC) to facilitating efficient entry into elongation or promoter escape (reviewed in [12–19]). Owing to its large size, its multisubunit composition, its conformational flexibility and the presence of numerous intrinsically disordered regions in many subunits [20,21], it remains very challenging to determine the complete structure of Mediator at high resolution. Initial EM investigations of negatively stained Mediator preparations provided outlines of the overall architecture of the complex as well as of the Mediator–Pol II holoenzyme complex at low resolution. These studies identified different modules within Mediator, referred to as Head, Middle and Tail in yeast [22–24] and initially named Head, body and leg in humans [25]. Mediator complexes can be isolated at least as two distinct stable entities containing or lacking the 4-subunit cyclin-dependent kinase 8 (CDK8) kinase module [25]. Over the past two decades, particularly in the last couple of years, remarkable progresses have been made in understanding structure–function relationships for Mediator as well as its role in the PIC, especially in yeast (reviewed in [26–30]). Here, we review recent insights regarding the Mediator complex structure and place them in historical perspective (Figure 1 and Supplementary Table S1).

## Structural studies of Mediator

The modular and dynamic nature of Mediator was pointed out from initial low-resolution EM reconstructions of endogenous complexes [22,25]. Early biochemical and genetic studies revealed many key subunit–subunit interactions that were instrumental to guide initial structural studies [31–41]. During the 2000s, high-resolution structures of yeast Mediator became available for the Med7C/Med21 heterodimer [42] (Figure 1A), the Med8C/Med18/Med20 subcomplex [43,44] (Figure 1B), the Med7N/Med31 submodule [45] and the Med11/Med22 four-helix bundle domain [46] (reviewed in [28]). These structures, although important, were limited to small parts of the whole complex. More recently, developments in the structural analysis of Mediator made high-resolution structural information of large parts of the entire complex a reality [47–50].

### Head module

The Mediator Head module is composed of seven highly conserved subunits (Med6, Med8, Med11, Med17, Med18, Med20 and Med22) and together with the Middle module, it plays an essential role during the PIC assembly, contacting the RNA Pol II and stabilizing its interaction with the general transcription factors (GTFs) (reviewed in [18]). The first major breakthrough in the structural characterization of the Head module occurred in 2006 with the successful production in insect cells of a recombinant 7-subunit complex from *Saccharomyces cerevisiae* [51] that enabled a first negative stain EM analysis [52] and, 5 years later, the determination of its crystal structure at 4.3 Å resolution [53]. The Head module structure has a characteristic shape reminiscent of a wrench constituted by three major domains that were initially named neck, fixed and movable jaws [51–53] (Figure 1C). These features were later confirmed by the structure of the *S. cerevisiae* Head module in complex with a 35-residues peptide containing five CTD heptad repeats [54] and by the crystal structure of the related *S. pombe* Head module at 3.4 Å resolution [55] (Figure 1D). This later structure led to a revised and more complete architecture of the Head, which has been described overall by eight distinct elements [55] (Supplementary Figure S1). One remarkable feature of the Head structure is the neck domain that stabilizes the module by forming a multi-helical bundle involving five different subunits, Med6, Med8, Med11, Med17 and Med22 [53–55] (Figure 1C). Importantly, these studies underline the fact that, when expressed individually, recombinant Mediator subunits are generally insoluble and stress the strict requirement for co-expression of subunits constituting a module or a submodule [31,42–46,51–53,56] (reviewed in [28]).

The structure determination of the Mediator Head module [53–55] represented a first major accomplishment towards the characterization of the entire Mediator complex at high resolution. In this context, information about the exact subunit localization and boundaries of the full three main modules was mandatory but remained rather elusive, even contradictory, more than 10 years after their first identification. Indeed at that time, the quality of the available EM structures was limited, as well as the capacity to analyze Mediator samples displaying considerable compositional and/or conformational heterogeneity. In 2014, two pivotal studies [57,58] reported cryo-electron microscopy (cryo-EM) analyses that completely redefined the modular organization of Mediator. Until then the Head module was assigned to one end of the Mediator structure, with the Middle and Tail modules folded one over the other to form the opposite portion of Mediator (reviewed in [59]). Using either tagged or deleted individual subunits combined with unequivocal docking of the Head module X-ray structure, these two studies delivered an improved cryo-EM reconstruction of yeast Mediator at 20–40 Å resolution [57,58] (Figure 1F) that showed, in a dramatic topsy-turvy twist, that the Head and Middle modules were forming the portion of Mediator previously attributed to the Middle and Tail modules, while the large opposite domain corresponded to the Tail, and not to the Head. As a consequence of this revised organization, EM structures of Mediator reported before 2014 should be interpreted with caution. A low-resolution map of human Mediator complex (Figure 1F) was also obtained [57], confirming the overall structural similarity of yeast and human Mediator despite their evolutionary divergence [24]. Previously unassigned metazoan subunits were also clearly localized. For example, Med27, Med28, Med29 and Med30 make extensive contacts with the Head module while Med26 associates with the Middle module [57]. Finally, a recombinant human 8-subunit Head module that additionally contained the metazoan subunit Med30 was successfully reconstituted in insect cells in 2014 [60]. The reconstitution of the recombinant human Head module was done in the context of an assembly of a functional 15-subunit human core Mediator complex that comprises the Head and Middle modules held together by the Med14 subunit (see below) [60]. However, structural data regarding the human Head module are still missing.

### Middle module

The Middle module comprises up to nine subunits (Med1, Med4, Med7, Med9, Med10, Med19, Med21 and Med31 as well as Med26 in mammals). The structure of the Middle module remained unknown for a long time and detailed structural information in yeast was limited until 2017 to two small subcomplexes, Med7N/Med31 [45] and Med7C/Med21 [42] (Figure 1A). The heterologous co-expression strategy was again successfully used in 2010 by the Cramer laboratory to purify a recombinant yeast 7-subunit Middle module [61]. In accordance with a previous report [38], an endogenous 7-subunit Middle module had been purified from a Δ*med19* strain, and Med19 was thus not included for the subsequent expression of this recombinant Mediator Middle module [61]. Limited proteolysis and co-expression pull-down experiments [61] together with previously published data [32–34,42,62], established at that time the most detailed protein interaction map of the Middle module. However, the high intrinsic flexibility of the module prevented its crystallization. Three years later, bacterial co-expression of a 6-subunit Middle module lacking Med1 combined with cross-linking experiments and homology models, allowed to propose a three-dimensional model of the Middle module [63]. In parallel, reconstitution in insect cells of a human recombinant Mediator 5-subunit Middle module (Med4, Med7, Med10, Med21 and Med31) was also obtained in 2014 [60]. Again, like for the human Head module, no structural data have yet emerged from these studies for the human Middle module. The only known structure of a human Mediator Middle module subunit is the N-terminal domain of Med26 [64] that serves in mammals as an overlapping docking site for super-elongation complexes (SECs) containing ELL/EAF family members as well as TFIID [65].

### Core Mediator

Core Mediator, a term that in the past has been used in different contexts, refers now to the minimal set of recombinant Mediator subunits active in transcription, which corresponds to the Head and Middle modules [48,60]. Indeed with the high-resolution structure of the Head module [53–55] and the three-dimensional architectural model of a 6-subunit Middle module [63] at hand in 2013, the next challenge was to decipher their spatial arrangement at the atomic level. But to do so, it appeared that one central piece of the puzzle was still missing. Med14 is one of the largest Mediator subunits and was assigned initially to either the Tail or the Middle module [24,33,38–40]. However, biochemical analyses of Mediator subassemblies in yeast have revealed very early on [35,36,40] that Med14 was tightly associated not only with Tail subunits (Med2, Med3, Med15, Med16) but also with many subunits of the Middle module (Med1, Med4, Med7, Med9 and Med21), suggesting that Med14 was possibly linking different modules. The role of Med14 as a key Mediator scaffold protein was confirmed in 2014 when the improved cryo-EM reconstruction of yeast Mediator at a resolution of 18 Å came out [57,58] (Figure 1F). In particular, C-terminal MBP tagging and N-terminal antibody labeling of Med14 [57] coupled with cross-linking and mass spectrometry of native Mediator [66] clearly indicated that the Med14 N and C termini were located at opposite ends of a large central density spanning ∼220 Å and connecting all three modules. Importantly, the scaffold function of yeast Med14 appeared to be conserved in the human Mediator complex [60].

**Figure 2.**
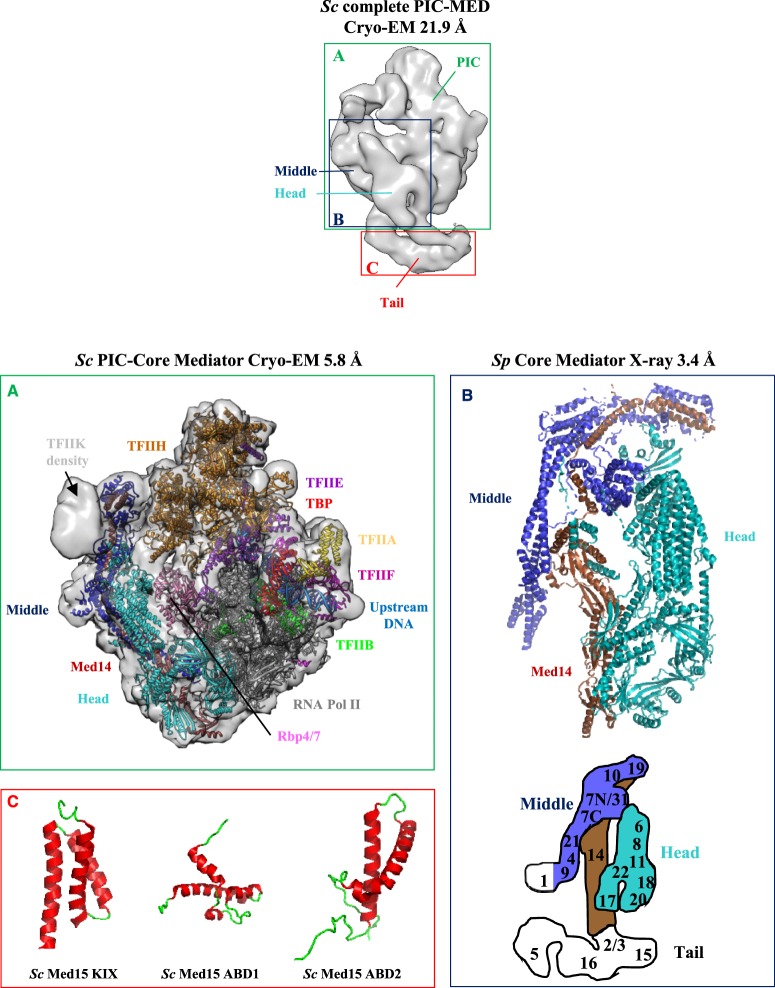
Structural studies of yeast Mediator complex. Cryo-EM three-dimensional reconstruction of a complete *S. cerevisiae* Mediator–RNA Pol II preinitiation complex (Sc PIC-MED) at 21.9 Å resolution (EMD-8308 [69]). PIC, Mediator Head, Middle and Tail modules are indicated. Individual panels display high-resolution structures of ((**A**), in green) *S. cerevisiae* PIC-core Mediator at 5.8 Å resolution (EMD-3850 [49]) in semi-transparent rendering with the docked atomic model (PDB 5OQM) in ribbon representation. The cryo-EM density for the mobile Kin28–Ccl1 kinase complex of TFIIH (TFIIK) is indicated. ((**B**), in dark blue) *S. pombe* core Mediator structure at 3.4 Å resolution (PDB 5N9J [47]). For the sake of simplicity, the Mediator Head module is depicted in cyan and the Middle module in blue, with their respective subunits displayed in ribbon representation. The Med14 subunit is in brown. For a more detailed description of core Mediator structure and its 13 defined submodules, see Supplementary Figure S1. The localization of Mediator subunits is schematized below in the Mediator diagram adapted from [50,57] with the same color code (Head module in cyan, Middle module in blue and Tail module in white). The Middle module Med1 subunit was included in recombinant core Mediator but is lacking in the crystal structure [47]. Med1 is thus colored in white. ((**C**), in red) NMR structures of *S. cerevisiae* Med15 KIX domain (PDB 2K0N [77]), Med15 ABD1 domain (PDB 2LPB [74]) and Med15 ABD2 domain (PDB 6ALY [78]).

The Head–Middle module interface, the precise localization of Med14 and the individual Middle module subunits were revealed soon after when two cryo-EM structures were reported, that of *S. cerevisiae* core Mediator bound to a core initiation complex (cITC–cMed that contains 31 polypeptides) at 9.7 Å resolution [48] (Figure 1G) and that of *S. pombe* Mediator at 4.4 Å resolution [50] (reviewed in [26,27,29]). The resulting architectural model also revealed the precise location of Mediator on RNA Pol II (see below). Finally, in a long-term effort to solve the high-resolution Mediator structure, the Cramer laboratory reported in 2017 the crystal structure of a 15-subunit core Mediator (cMed) from *S. pombe* at an impressive 3.4 Å resolution [47] (Figures 1H, 2 and Supplementary Figure S1). The cMed structure is divided into 13 submodules, 8 in the Head as previously defined [55] and 5 in the Middle module (Supplementary Figure S1). Especially noteworthy are the tether regions formed by the C-terminus of Med6 and the N-terminus of Med17 that both associate with Med14 linking the Head and Middle modules [47] and the key architectural roles performed by Med14 and Med17 that make an extensive network of intersubunit interactions, with Med14 connecting all three modules [47,48,50,60,66]. Thus, these structures represent a major advance in our understanding of the structure of yeast core Mediator and overall, only Med1 remains unresolved [47,50].

### Tail module

The Tail module subunits are the most evolutionarily divergent [9] and include in *S. cerevisiae* Med2, Med3, Med5, Med15 and Med16. In addition to the conserved Med15 and Med16, Mediator Tail includes in metazoans three other subunits, Med23, Med24 and Med25 with Med24 considered to be a highly divergent ortholog of yeast Med5 [9] (reviewed in [67]). It has also been suggested that metazoans Med27 and Med29 are very distant orthologs of yeast Med3 and Med2, respectively [9]. These subunits are thus now often referred to in the literature as Med2/29, Med3/27 and Med5/24. Their precise assignment along with the two other metazoan subunits Med28 and Med30 remains to be assessed. Notably, Med27, Med28, Med29 and Med30 make extensive contacts with the Head module [57,60,68] but in *S. pombe* there is also a clear Med27-Tail connection [50]. As mentioned above, the Med14 subunit makes extensive contacts with all three modules [47–50,57,60,66] and is now viewed as a key architectural backbone of the Mediator complex rather than simply a Tail and/or a Middle subunit.

**Figure 3.**
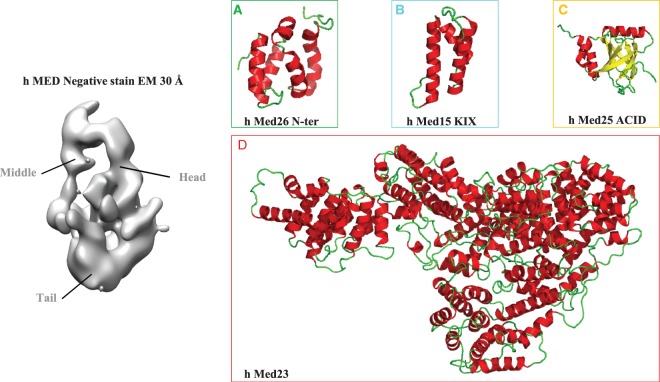
Structural studies of human Mediator complex. EM map of Human Mediator at 30 Å resolution (EMD-2635 [57]). Mediator Head, Middle and Tail modules are indicated. Individual panels display high-resolution structures of ((**A**), in green) the N-terminal domain of Human Med26 (PDB 5ODD [64]), ((**B**), in cyan) KIX domain of human Med15 (PDB 2GUT [80]), ((**C**), in yellow) ACID/PTOV domain of human Med25 (PDB 2L23, 2XNF, 2L6U and 2KY6 [73,75,76,79]) and ((**D**), in red) human Med23 (PDB 6H02 [72]). Med26 associates with the Middle domain via interactions with Med4, Med7 and Med19 [57]. Note that Med26 N- and C-terminal domains support interactions with TFIID and transcription elongation factors and with Mediator complex, respectively [65]. Med15 and Med23 have been assigned to the Tail module and Med25, which engages in an extensive network of interactions with other Tail subunits is believed to be a component of the Tail [57]. For details, refer to the text.

Notwithstanding, the Tail module remains currently unresolved likely due to its conformational heterogeneity (reviewed in [26]). For example, in a small subset of the *S. cerevisiae* Mediator–PIC particles [69] (Figure 2), alternate Tail module conformations were observed consistent with the previous observation that the presence of the yeast transcription factor Gcn4 causes a rotation of the Tail module [57]. These conformational changes triggered by transcription factors (within the limits of the very early low-resolution EM reconstructions) are also evident in the human Mediator complex [25,70,71]. Such structural transitions, which seem to propagate throughout the entire Mediator complex, have been hypothesized to promote its stable association with the transcription machinery (reviewed in [12]). These observations raise the possibility that transcription factors capable of coordinating changes in Mediator conformation required for its interaction with RNA Pol II could play major roles in governing transcription. An intriguing possibility is that the Tail module may exert an inhibitory effect on the Head and/or Middle modules in order to prevent promiscuous transcription in absence of activators. Anyhow, until 2018 when the first structure of a whole Mediator Tail subunit (human Med23) was finally solved [72], only small portions of subunits from the Tail module were structurally characterized [73–80] (Figures 1I, 2 and 3). Despite this limited structural information, the overall architecture and subunits interactions are now fairly well characterized, at least in yeast [57,66]. Consistent with early observations that a C-terminal truncation of Med14 caused the loss of Tail subunits [40] and that Med2/Med3/Med15 form a stable subcomplex [31,81], Med2, Med3, Med5, Med15 and Med16 form the bottom domain of the structure and are interconnected to the rest of Mediator through the C-terminus of Med14 [50,57,66]. Importantly, this structural architecture appears to be conserved in the human Mediator complex where Med14 is able to bridge the Head, Middle and Tail modules [60]. However, the human Tail module constitutes a very complex interaction hub and low-resolution Tail EM density of human Mediator at 30 Å resolution appeared much larger that its yeast counterpart [57] (Figure 3). Although the precise metazoan Tail subunit organization has yet to be determined, Med16, Med23 and Med24 are known to form a submodule [82,83]. The reconstitution of a 15-subunit human core Mediator complex [60] and the recent crystal structure of human Med23 [72] are elements that should progressively pave the way for future reconstruction of the full complex architecture.

Finally, several studies identified Mediator subunits that are contacted by transcription factors and these interactions tend to cluster in the Tail module (reviewed in [13]). Structural analysis using mainly nuclear magnetic resonance (NMR) has revealed the molecular details of interactions between transactivation domains (TAD) of transcription factors and the activator-binding domains (ABD) of a few Mediator Tail subunits including sterol regulatory element-binding protein (SREBP)/Med15 [80], VP16/Med25 [73,76,79], the PEA3 subfamily of Ets transcription factors/Med25 [84,85], ATF6α/Med25 [86] and Gcn4/Med15 [74,78] (Figures 2 and 3). These studies illustrate the conformational heterogeneity of intrinsically unstructured TADs that are engaged in multiple low-affinity and low-specificity interactions with their targets (reviewed in [87]). These multivalent interactions may drive phase-separated condensates that are potentially important for transcriptional control [88–91]. These structural studies are also particularly relevant for human diseases (reviewed in [92]) and the development of small molecules to interfere with transcription factor–Mediator subunit interactions (reviewed in [93]).

### CDK8 kinase module

A major point of agreement established long ago is that Mediator subunits are organized into three modules (Head, Middle, Tail) and a four-subunit (CDK8, CycC, Med12 and Med13) dissociable CDK8 kinase module (CKM) [94]. The kinase module has been associated primarily with repressive functions on Mediator but has also been implicated in activation of transcription (reviewed in [19]). Multiple studies have indicated that the RNA Pol II and the CKM interact with the Mediator complex in a mutually exclusive manner [95–99] and that ejection of the kinase module is required for Mediator to join the PIC (reviewed in [27]). The structure of *S. pombe* CycC [100] and of human CDK8/CycC [101] have been solved but limited information is available for Med12 or Med13. The overall molecular architecture of budding yeast CKM has been obtained in 2013 by cryo-EM at ∼15 Å resolution [99,102] (Figure 1E). Collectively, these two studies demonstrated that Med13 and the CDK8/CycC pair are at opposite end of the CKM structure, connected by Med12 that forms the central lobe [99,102]. Interestingly, the interaction between Mediator and CKM appears very similar in yeast and human [96] Mediators, with the CKM tethered via its Med13 subunit to the hook domain formed by Middle module subunits at the very end of Mediator structure (reviewed in [26,27]). An extended interface with Mediator that overlaps with the position that would be occupied by the RNA pol II holoenzyme was also observed in yeast [95,99], suggesting that Mediator and CKM may interact in different ways. Structural data with human Mediator complex rather suggest that CKM-induced structural rearrangements block Mediator–RNA Pol II interaction, or vice versa [96,103]. The structural basis of this CKM inhibitory interaction awaits a high-resolution structure of the entire Mediator complex with its kinase module.

## Structural studies of RNA Pol II–Mediator complexes

Because RNA Pol II and Mediator lie at the center of the transcription machinery, several studies have aimed to characterize the structure of the Mediator–RNA Pol II assembly [22–24,48–50,52,57,58,69,99,103–105] (Supplementary Table S1). Initial EM studies led to inconsistent locations of Mediator on RNA Pol II, probably owing to Mediator heterogeneity and low-resolution reconstruction. Mediator density in yeast was observed at four different locations on RNA Pol II [23,53,54,99,104–106] and a fifth position was obtained in the human RNA Pol II–TFIIF–VP16–Mediator complex [103]. Recent low- [69] and high-resolution cryo-EM structures of *S. cerevisiae* [48,49] (Figure 2) and *S. pombe* holoenzymes [50] have been reported and show similar overall structures with subtle differences in detail, resolving previous ambiguities. In these structures, Med18 and Med20 contact the RNA Pol II Rpb3–Rpb11 heterodimer and the TFIIB β-ribbon, Med22 and Med8 contact Rpb4 and Med9 contacts the RNA Pol II foot region [49]. The structural comparison of both unbound Mediator [47] and cITC–cMed [48] with the latest available PIC–cMed cryo-EM structures [49,50,107] suggests a significant repositioning of the Middle module with respect to the Head module upon PIC binding. In particular, four submodules undergo concerted movements owing to large conformational changes in Med14 and inherent structural flexibility of the Med7/Med21 hinge (Supplementary Figure S1). Collectively, these Mediator–Pol II contacts are globally consistent with functional data (reviewed in [18]) but do not exclude the possibility that other conformations could exist during the assembly of the PIC *in vivo* [108,109].

These extensive core Mediator–RNA Pol II contacts may appear paradoxical given that phosphorylation of the unstructured Pol II CTD is known to drive dissociation of Mediator from RNA Pol II [110–113]. However, to date, it remains unclear how the destabilization of the complex occurs, especially since the path of the CTD along Mediator is still under debate [48–50,54,69]. The recent PIC–cMed cryo-EM structures [48,49] showed evidence that the linker to the mobile CTD extends from RNA Pol II towards the inner surface of a ‘cradle’ formed between RNA Pol II and Mediator to reach the TFIIH kinase (reviewed in [26]). An intriguing possibility is that the global structural organization of Mediator defines the topological and mobility restraints of the entire PIC assembly to properly orient the RNA Pol II CTD for efficient phosphorylation by TFIIH. Phosphorylation of CTD would result in the accumulation of negative charges and thus lead to dissociation of Mediator [49].

## Conclusion and perspectives

From the averaging of 54 individual particles in negative stain in 1999 to the highest resolution structures of core Mediator alone and within the PIC determined in 2017 by modern cryo-EM single particle reconstruction techniques, nearly 20 years have passed. During this period, the revolution of integrated structural biology irrigated the studies devoted to the Mediator and allowed unprecedented progress in our quest to reveal its structure that could not have been anticipated or even dreamed ∼10 years ago. While the structures of the Head and Middle modules have been revealed, little structural information is still available for the Tail and CKM and their high-resolution structural determination constitutes one of the next challenges. In addition, the structural impact of the association of the PIC with Mediator and transcription factors remains elusive. It seems now possible, within the next decade, to reveal the structure of the full Mediator complex, both in yeast and human. In a mid-term effort, it should be possible to directly visualize how transcription factors influence the Mediator Tail module structure, how the regulatory signals are transmitted to the core Mediator and subsequently to the PIC, and how the kinase module interferes with Pol II binding. Finally, near-atomic resolution cryo-EM maps of the PIC complex including TFIIH and the Mediator Head and Middle modules already represent major breakthroughs towards these goals and together with the very recent cryo-EM structures of yeast [114] and human [115] TFIID, provide an exciting framework which paves the way for the long awaited structural characterization of the entire initiation machinery.

## Abbreviations

ACID/PTOV: activator-interacting domain/prostate overexpressed
CDK8: cyclin-dependent kinase 8
cITC: core initiation complex
CKM: CDK8 kinase module
cMed: core Mediator
cryo-EM: cryogenic electron microscopy
CTD: carboxy-terminal domain
EM: electron microscopy
GTF: general transcription factor
KIX: kinase-inducible domain interacting domain
MED: Mediator
PIC: PreInitiation complex
RNA Pol II: RNA polymerase II
Srb: suppressor of RNA polymerase B
TAD: transactivation domain
