# Supplementary material for: Twenty years of Mediator complex structural studies
Source: Biochem Soc Trans. 2019 Feb 7;47(1):399–410. doi: 10.1042/BST20180608 (PMC6393861; doi:10.1042/BST20180608)
Supplement: Supplementary Table and Figure [file BST-47-399-s1.pdf]

Supplementary Table 1

Crystal, NMR and Electron Microscopy reconstructions of Mediator in chronological order

Module	Subunits	Org.[a]	Construct	Method	Expression	PDB/EMD	Resol. (Å)	Year	Reference
Head + Middle + Tail		*Sc*	native Mediator	EM	*Sc*	-	40	1999	(*1*)
Head + Middle + Tail		*Sc*	native Mediator[b]	EM	*Sc*	-	30-35	2000	(*2*)
Head + Middle + Tail		*Hs*	native from HeLa cells via SREBP-1a affinity resin (CRSP and ARC-L (with CKM))	EM	*Hs*	-	32	2002	(*3*)
Head + Middle + Tail		*Hs*	native from HeLa cells P1.0M fraction via GST-CTD	EM	*Hs*	-	32	2002	(*4*)
Head + Middle + Tail		*Hs*	native from HeLa cells P0.5M fraction (CRSP/Med2 lacking CKM, MED1 and MED26)	EM	*Hs*	-	31	2004	(*5*)
Head + Middle + Tail		*Hs*	native from HeLa cells P1.0M fraction - TR and VDR	EM	*Hs*	-	29	2004	(*6*)
Middle	7/21	*Sc*	7C(102-205)/21(1-132)	X-ray	*E. Coli*	1YKH	3.0	2005	(*7*)
Kinase	CycC	*Sp*	CyC(5-228)	X-ray	*E. Coli*	1ZP2	3.0	2005	(*8*)
Head	8/18/20	*Sc*	8C(190-210)/18(Δ109-140)/20	X-ray	*E. Coli*	2HZS	2.7	2006	(*9*)
Head	18/20	*Sc*	18(Δ109-140)/20	X-ray	*E. Coli*	2HZM	2.4	2006	(*9*)
Tail	15	*Hs*	15(5-78) KIX domain	NMR	*E. Coli*	2GUT	-	2006	(*10*)
Head + Middle + Tail + Kinase		*Sp*	native Mediator (S-Mediator) and native Mediator with CKM (L-Mediator)	EM	*Sp*	-	25	2006	(*11*)
Head + Middle + Tail		*Hs*	native from HeLa cells + CDK8 module + TRAPP + GCN5L (T/G-Mediator)	EM	*Hs*	-	-	2008	(*12*)
Tail	15	*Sc*	15(6-90) KIX domain	NMR	*E. Coli*	2K0N	-	2008	(*13*)
Head	8/18	*Sp*	8C(180-200)/18	X-ray	*E. Coli*	3C0T	2.4	2008	(*14*)
Middle	7/31	*Sc*	7N(1-83)/31	X-ray	*E. Coli*	3FBI	2.8	2009	(*15*)
Kinase	12/13/CDK8/CycC	*Hs*	12/13/Glu-tagged CDK8/CycC	EM	Baculo	-	38	2009	(*16*)
Head + Middle + Tail		*Sc*	native Mediator harboring Med8-3xHA and Med22-10xHis	CryoEM	*Sc*	-	28	2009	(*17*)
Head	6/8/11/17/18/20/22	*Sc*	6/8/11/17/18/20/22	EM	Baculo	-	30-35	2010	(*18*)
Head + Middle + Tail		*Hs*	native Mediator from HeLa nuclear extract + p53 activation domain	EM	*Hs*	-	34	2010	(*19*)
Head	11/22	*Sc*	11(1-89)/22(5-89)	X-ray	*E. Coli*	3R84	2.1	2011	(*20*)
Head	6/8/11/17/18/20/22	*Sc*	6/8/11(Δ1-16)/17(109-687)/18(Δ109-140)/20/22	X-ray	Baculo	3RJ1	4.3	2011	(*21*)
Tail	15	*Sc*	15(158-238) ABD1 in complex with Gcn4 activation domain (101-134)	NMR	*E. Coli*	2LPB	-	2011	(*22*)
Tail	25	*Hs*	25(391-548) ACID/PTOV domain and characterization of its binding site for VP16 activation domain	NMR	*E. Coli*	2L23, 2XNF, 2L6U, 2KY6	-	2011	(*23-26*)
Kinase	CDK8/CycC	*Hs*	CDK8(1-403)/CyC(1-283)	X-ray	Baculo	3RGF[c]	2.2	2011	(*27*)
Holoenzyme		*Hs*	native Mediator from HeLa nuclear extract + RNAPII + TFIIF + VP16	CryoEM	*Hs*	EMD 5343	36	2011	(*28*)
Head	6/8/11/17/18/22	*Sp*	6(1-180)/8/11/17(77-545)/18/22[d]	X-ray	*E. Coli*	4H63	3.4	2012	(*29*)

Head	6	*Sp*	6(9-180)	X-ray	*E. Coli*	4H61	2.7	2012	(*29*)
Head	11/17/22	*Sc*	11C(84-115)-22C(96-121)[e]/17C(377-687)	X-ray	*E. Coli*	4H62	3.0	2012	(*29*)
Head	6/8/11/17/18/20/22	*Sc*	native head module harboring 8-TAP/Δ16 + 35-residues peptide (5 CTD repeats)	X-ray	*Sc*	4GWP	4.2	2012	(*30*)
Head + RNAPII	6/8/11/17/18/20/22	*Sc*	Head + RNAPII + TFIIF + TFIIB + TBP + DNA[f]	cryoEM	Mixed	EMD-5407	16	2012	(*31*)
Holoenzyme		*Hs*	native Mediator from HeLa nuclear extract + RNAPII + TFIIF	CryoEM	*Hs*	EMD-5344	32	2012	(*32*)
Kinase	12/13/CDK8/CycC	*Sc*	native CKM module harboring CDK8-TAP	cryoEM	*Sc*	EMD-5588	15	2013	(*33*)
Kinase	12/13/CDK8/CycC	*Sc*	native CKM module harboring CDK8-TAP or Med12-TAP	EM	*Sc*	-	30-35	2013	(*34*)
Head + Middle + Tail		*Sc*	native Mediator harboring 10xHis tag at the C-ter of Med14, 17, 22 or 21	EM	*Sc*	-	32-38	2014	(*35*)
Head + Middle + Tail		*Sc*	native Mediator harboring Med16-MBP and CBP-Med5	CryoEM	*Sc*	EMD-2634	18	2014	(*36*)
Head + Middle + Tail		*Hs*	native Mediator from Hela-S3 cells stably expressing Flag-Med26	EM	*Hs*	EMD-2635	30	2014	(*36*)
Head + Middle + RNAPII	Head/Middle/14/19	*Sc*	Head + Middle + 19 + 14(1-745) + RNAPII + TBP + TFIIB + TFIIF	cryoEM	*Sc/E. Coli*	EMD-2786, 4V1N, 4V1O	9.7	2015	(*37*)
Holoenzyme		*Sc*	native Mediator harboring Med21-Flag-ProteinA	EM	*Sc*	-	-	2016	(*38*)
Holoenzyme		*Hs*	native Mediator from HeLa cells stably expressing Flag-Med7	EM	*Hs*	-	-	2016	(*38*)
Holoenzyme		*Sc*	Mediator + RNAPII + TFIIA + TFIIB + TFIIH + TFIIE + TFIIF + TBP (52 polypeptides)	CryoEM	*Sc*	EMD-8308, 5SVA	21.9	2016	(*39*)
Holoenzyme		*Sp*	native Mediator harboring Med7-3xFlag ΔMed13[g] + RNAPII	CryoEM	*Sp*	EMD-8480, 5U0S	7.8	2017	(*40*)
Holoenzyme		*Sc*	Head/Middle/19/14(1-745) + RNAPII + TBP + TFIIA + TFIIB + TFIIE + TFIIF + TFIIH	CryoEM	Mixed	EMD-3850, 5OQM	5.8	2017	(*41*)
Middle	26	*Hs*	26(1-92)	NMR	*E. Coli*	5ODD	-	2017	(*42*)
Head + Middle	4/6/7/8/9/10/11/14/17/18/19/20/21/22/31	*Sp*	1/4/6/7/8/9/10/11(Δ112-116)/6his-14(Δ581-879)/17/18/19/20/21/22/31[h]	X-ray	*E. Coli*	5N9J	3.4	2017	(*43*)
Tail	15	*Sc*	15(277-368) ABD2	NMR	*E. Coli*	6ALY	-	2018	(*44*)
Tail	23	*Hs*	23(1-1334)[i]	X-ray	Baculo	6H02	2.8	2018	(*45*)

Supplementary Table 1 adapted from (*46*) with permission from Elsevier.

[a] Organism : Sc, *Saccharomyces cerevisiae* ; Sp, *Schizosaccharomyces pombe* ; Hs, *Homo sapiens*

[b] The 3D structures of murine and human Mediator were also reported. The overall architecture is conserved.

[c] Many structures were then reported in complex with small molecule inhibitors of CDK8 such as Cortistatin A (PDB 4CRL (*47*)).

[d] Med20 subunit is not present in the crystals but was unambiguously modelled and positioned by superimposing the previous *Sc* Med18/Med20 structure (*9*).

[e] Med11C/Med22C is a fusion construct with a linker (GAGSGAGSG) inserted between the C terminus of Med11 and residue 96 of Med22.

[f] unable to conclusively identify density corresponding to TFIIB, TBP and DNA. Consequently the cryo-EM map revealed the structure of Mediator Head + RNA polymerase II + TFIIF.

[g] Cryo-EM map was masked to hide the Tail. The atomic model includes 16 subunits (Med4/Med6/Med7/Med8/Med9/Med10/Med11/Med14/Med17/Med18/Med19/Med20/Med21/Med22/Med27/Med31).

[h] The core Mediator structure comprises 15 subunits (Head + Middle + Med14) and only lacks Med1 which probably dissociated during crystallization.

[i] The structure of Med23 was determined in complex with a stabilizing nanobody.

References

1. Asturias, F. J., Jiang, Y. W., Myers, L. C., Gustafsson, C. M., and Kornberg, R. D. (1999) Conserved structures of mediator and RNA polymerase II holoenzyme, *Science 283*, 985-987.
2. Dotson, M. R., Yuan, C. X., Roeder, R. G., Myers, L. C., Gustafsson, C. M., Jiang, Y. W., et al. (2000) Structural organization of yeast and mammalian mediator complexes, *Proc Natl Acad Sci U S A 97*, 14307-14310.
3. Taatjes, D. J., Näär, A. M., Andel, F., 3rd, Nogales, E., and Tjian, R. (2002) Structure, function, and activator-induced conformations of the CRSP coactivator, *Science 295*, 1058-1062.
4. Näär, A. M., Taatjes, D. J., Zhai, W., Nogales, E., and Tjian, R. (2002) Human CRSP interacts with RNA polymerase II CTD and adopts a specific CTD-bound conformation, *Genes Dev 16*, 1339-1344.
5. Taatjes, D. J., and Tjian, R. (2004) Structure and function of CRSP/Med2; a promoter-selective transcriptional coactivator complex, *Mol Cell 14*, 675-683.
6. Taatjes, D. J., Schneider-Poetsch, T., and Tjian, R. (2004) Distinct conformational states of nuclear receptor-bound CRSP-Med complexes, *Nat Struct Mol Biol 11*, 664-671.
7. Baumli, S., Hoeppner, S., and Cramer, P. (2005) A conserved mediator hinge revealed in the structure of the MED7.MED21 (Med7.Srb7) heterodimer, *J Biol Chem 280*, 18171-18178.
8. Hoeppner, S., Baumli, S., and Cramer, P. (2005) Structure of the mediator subunit cyclin C and its implications for CDK8 function, *J Mol Biol 350*, 833-842.
9. Larivière, L., Geiger, S., Hoeppner, S., Rother, S., Strasser, K., and Cramer, P. (2006) Structure and TBP binding of the Mediator head subcomplex Med8-Med18-Med20, *Nat Struct Mol Biol 13*, 895-901.
10. Yang, F., Vought, B. W., Satterlee, J. S., Walker, A. K., Jim Sun, Z. Y., Watts, J. L., et al. (2006) An ARC/Mediator subunit required for SREBP control of cholesterol and lipid homeostasis, *Nature 442*, 700-704.
11. Elmlund, H., Baraznenok, V., Lindahl, M., Samuelsen, C. O., Koeck, P. J., Holmberg, S., et al. (2006) The cyclin-dependent kinase 8 module sterically blocks Mediator interactions with RNA polymerase II, *Proc Natl Acad Sci U S A 103*, 15788-15793.
12. Meyer, K. D., Donner, A. J., Knuesel, M. T., York, A. G., Espinosa, J. M., and Taatjes, D. J. (2008) Cooperative activity of cdk8 and GCN5L within Mediator directs tandem phosphoacetylation of histone H3, *Embo J 27*, 1447-1457.
13. Thakur, J. K., Arthanari, H., Yang, F., Pan, S. J., Fan, X., Breger, J., et al. (2008) A nuclear receptor-like pathway regulating multidrug resistance in fungi, *Nature 452*, 604-609.
14. Larivière, L., Seizl, M., van Wageningen, S., Rother, S., van de Pasch, L., Feldmann, H., et al. (2008) Structure-system correlation identifies a gene regulatory Mediator submodule, *Genes Dev 22*, 872-877.
15. Koschubs, T., Seizl, M., Larivière, L., Kurth, F., Baumli, S., Martin, D. E., et al. (2009) Identification, structure, and functional requirement of the Mediator submodule Med7N/31, *Embo J 28*, 69-80.

16. Knuesel, M. T., Meyer, K. D., Bernecky, C., and Taatjes, D. J. (2009) The human CDK8 subcomplex is a molecular switch that controls Mediator coactivator function, *Genes Dev 23*, 439-451.
17. Cai, G., Imasaki, T., Takagi, Y., and Asturias, F. J. (2009) Mediator structural conservation and implications for the regulation mechanism, *Structure 17*, 559-567.
18. Cai, G., Imasaki, T., Yamada, K., Cardelli, F., Takagi, Y., and Asturias, F. J. (2010) Mediator head module structure and functional interactions, *Nat Struct Mol Biol 17*, 273-279.
19. Meyer, K. D., Lin, S. C., Bernecky, C., Gao, Y., and Taatjes, D. J. (2010) p53 activates transcription by directing structural shifts in Mediator, *Nat Struct Mol Biol 17*, 753-760.
20. Seizl, M., Larivière, L., Pfaffeneder, T., Wenzeck, L., and Cramer, P. (2011) Mediator head subcomplex Med11/22 contains a common helix bundle building block with a specific function in transcription initiation complex stabilization, *Nucleic Acids Res 39*, 6291-6304.
21. Imasaki, T., Calero, G., Cai, G., Tsai, K. L., Yamada, K., Cardelli, F., et al. (2011) Architecture of the Mediator head module, *Nature 475*, 240-243.
22. Brzovic, P. S., Heikaus, C. C., Kisselev, L., Vernon, R., Herbig, E., Pacheco, D., et al. (2011) The acidic transcription activator gcn4 binds the mediator subunit gal11/med15 using a simple protein interface forming a fuzzy complex, *Mol Cell 44*, 942-953.
23. Bontems, F., Verger, A., Dewitte, F., Lens, Z., Baert, J. L., Ferreira, E., et al. (2011) NMR structure of the human Mediator MED25 ACID domain, *J Struct Biol 174*, 245-251.
24. Eletsky, A., Ruyechan, W. T., Xiao, R., Acton, T. B., Montelione, G. T., and Szyperski, T. (2011) Solution NMR structure of MED25 (391-543) comprising the activator-interacting domain (ACID) of human mediator subunit 25, *J Struct Funct Genomics 12*, 159-166.
25. Milbradt, A. G., Kulkarni, M., Yi, T., Takeuchi, K., Sun, Z. J., Luna, R. E., et al. (2011) Structure of the VP16 transactivator target in ARC/Mediator, *Nat Struct Biol 18*, 410-415.
26. Vojnic, E., Mourao, A., Seizl, M., Simon, B., Wenzeck, L., Larivière, L., et al. (2011) The mediator MED25 activator interaction domain: structure and cooperative binding of VP16 subdomains, *Nat Struct Biol 18*, 404-409.
27. Schneider, E. V., Bottcher, J., Blaesse, M., Neumann, L., Huber, R., and Maskos, K. (2011) The Structure of CDK8/CycC Implicates Specificity in the CDK/Cyclin Family and Reveals Interaction with a Deep Pocket Binder, *J Mol Biol 412*, 251-266.
28. Bernecky, C., Grob, P., Ebmeier, C. C., Nogales, E., and Taatjes, D. J. (2011) Molecular architecture of the human Mediator-RNA polymerase II-TFIIF assembly, *PLoS Biol 9*, e1000603.
29. Larivière, L., Plaschka, C., Seizl, M., Wenzeck, L., Kurth, F., and Cramer, P. (2012) Structure of the Mediator head module, *Nature 492*, 448-451.
30. Robinson, P. J., Bushnell, D. A., Trnka, M. J., Burlingame, A. L., and Kornberg, R. D. (2012) Structure of the mediator head module bound to the carboxy-terminal domain of RNA polymerase II, *Proc Natl Acad Sci U S A 109*, 17931-17935.
31. Cai, G., Chaban, Y. L., Imasaki, T., Kovacs, J. A., Calero, G., Penczek, P. A., et al. (2012) Interaction of the mediator head module with RNA polymerase II, *Structure 20*, 899-910.

32. Bernecky, C., and Taatjes, D. J. (2012) Activator-mediator binding stabilizes RNA polymerase II orientation within the human mediator-RNA polymerase II-TFIIF assembly, *J Mol Biol 417*, 387-394.
33. Tsai, K. L., Sato, S., Tomomori-Sato, C., Conaway, R. C., Conaway, J. W., and Asturias, F. J. (2013) A conserved Mediator-CDK8 kinase module association regulates Mediator-RNA polymerase II interaction, *Nat Struct Mol Biol 20*, 611-619.
34. Wang, X., Wang, J., Ding, Z., Ji, J., Sun, Q., and Cai, G. (2013) Structural flexibility and functional interaction of mediator Cdk8 module, *Protein Cell 4*, 911-920.
35. Wang, X., Sun, Q., Ding, Z., Ji, J., Wang, J., Kong, X., et al. (2014) Redefining the modular organization of the core Mediator complex, *Cell Res 24*, 796-808.
36. Tsai, K. L., Tomomori-Sato, C., Sato, S., Conaway, R. C., Conaway, J. W., and Asturias, F. J. (2014) Subunit Architecture and Functional Modular Rearrangements of the Transcriptional Mediator Complex, *Cell 157*, 1430-1444.
37. Plaschka, C., Larivière, L., Wenzeck, L., Seizl, M., Hemann, M., Tegunov, D., et al. (2015) Architecture of the RNA polymerase II-Mediator core initiation complex, *Nature 518*, 376-380.
38. Sato, S., Tomomori-Sato, C., Tsai, K. L., Yu, X., Sardiu, M., Saraf, A., et al. (2016) Role for the MED21-MED7 Hinge in Assembly of the Mediator-RNA Polymerase II Holoenzyme, *J Biol Chem 291*, 26886-26898.
39. Robinson, P. J., Trnka, M. J., Bushnell, D. A., Davis, R. E., Mattei, P. J., Burlingame, A. L., et al. (2016) Structure of a Complete Mediator-RNA Polymerase II Pre-Initiation Complex, *Cell 166*, 1411-1422 e1416.
40. Tsai, K. L., Yu, X., Gopalan, S., Chao, T. C., Zhang, Y., Florens, L., et al. (2017) Mediator structure and rearrangements required for holoenzyme formation, *Nature 544*, 196-201.
41. Schilbach, S., Hantsche, M., Tegunov, D., Dienemann, C., Wigge, C., Urlaub, H., et al. (2017) Structures of transcription pre-initiation complex with TFIIH and Mediator, *Nature 551*, 204-209.
42. Lens, Z., Cantrelle, F. X., Peruzzini, R., Hanoulle, X., Dewitte, F., Ferreira, E., et al. (2017) Solution Structure of the N-Terminal Domain of Mediator Subunit MED26 and Molecular Characterization of Its Interaction with EAF1 and TAF7, *J Mol Biol 429*, 3043-3055.
43. Nozawa, K., Schneider, T. R., and Cramer, P. (2017) Core Mediator structure at 3.4 Å extends model of transcription initiation complex, *Nature 545*, 248-251.
44. Tuttle, L. M., Pacheco, D., Warfield, L., Luo, J., Ranish, J., Hahn, S., et al. (2018) Gcn4-Mediator Specificity Is Mediated by a Large and Dynamic Fuzzy Protein-Protein Complex, *Cell Rep 22*, 3251-3264.
45. Monté, D., Clantin, B., Dewitte, F., Lens, Z., Rucktooa, P., Pardon, E., et al. (2018) Crystal structure of human Mediator subunit MED23, *Nat Commun 9*, 3389.
46. Larivière, L., Seizl, M., and Cramer, P. (2012) A structural perspective on Mediator function, *Curr Opin Cell Biol 24*, 305-313.
47. Pelish, H. E., Liau, B. B., Nitulescu, II, Tangpeerachaikul, A., Poss, Z. C., Da Silva, D. H., et al. (2015) Mediator kinase inhibition further activates super-enhancer-associated genes in AML, *Nature 526*, 273-276.

Supplementary Figure 1. Submodule architecture of core yeast Mediator. The 13 submodules which were defined in 2017 (*47*) are indicated and colored: 8 in the Head module (shoulder, arm, spine, finger, joint, movable jaw, tooth and nose) and 5 in the Middle module (hook, knob, connector, plank and beam). The hinge in the Med7C/Med21 subcomplex that flexibly links the hook to the connector is indicated (see also Figure 1A). The Middle module Med1 subunit was included in recombinant core Mediator but is lacking in the crystal structure (*47*). The second view is obtained by a -105° rotation around the z axis.

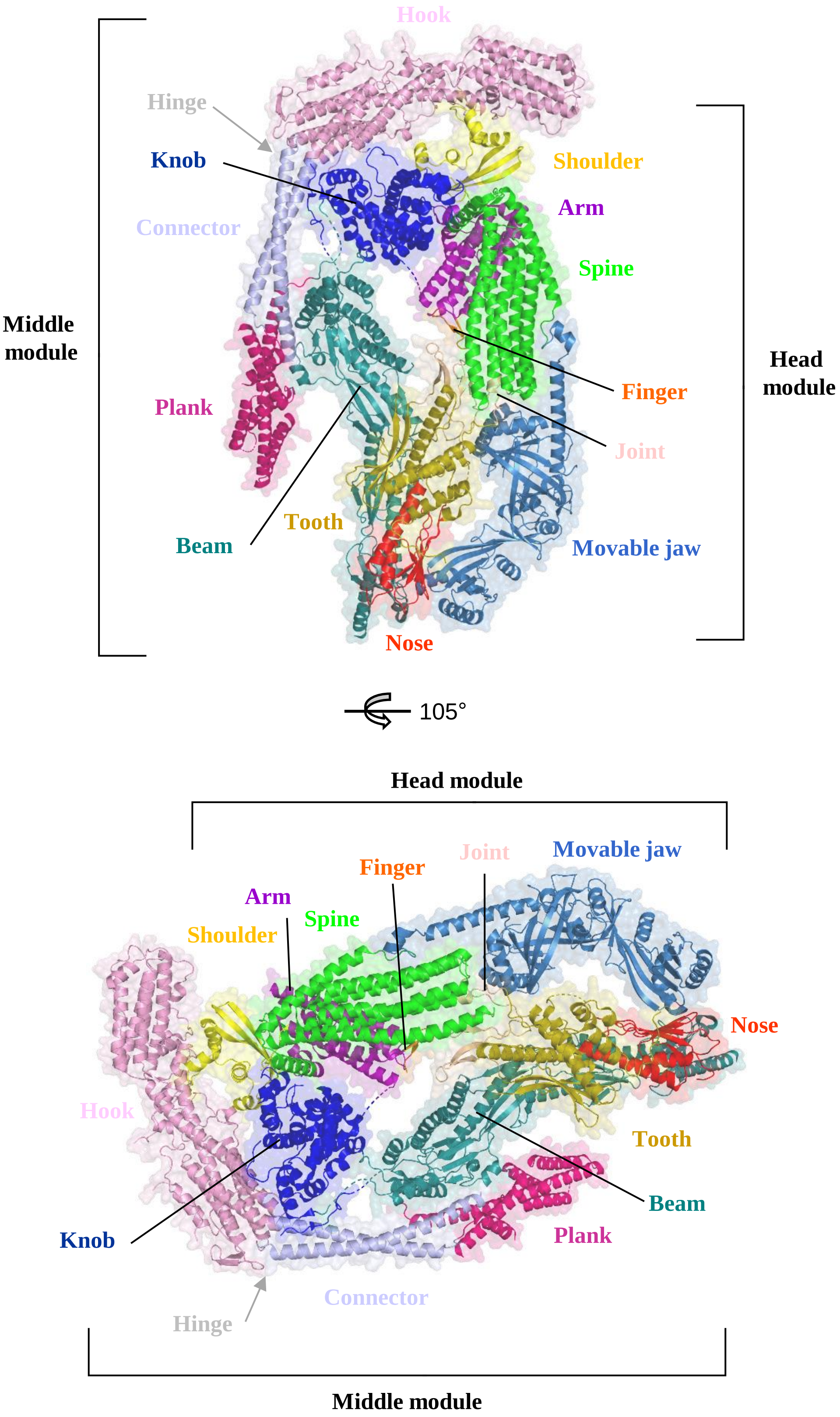

Supplementary Figure 1
